# Genetic Findings in Seven Cochlear Implanted Patients with Severe-to-Profound Hearing Loss

**DOI:** 10.3390/genes17080942

**Published:** 2026-08-12

**Authors:** Rieke Ollermann, Fei Song, Marta Owczarek-Lipska, Amilcar Perez-Riverol, Gregor Dombrowsky, Andreas Radeloff, John Neidhardt

**Affiliations:** 1 Human Genetics, Faculty of Medicine and Health Science, University of Oldenburg, 26129 Oldenburg, Germany; 2 Oto-Rhino-Laryngology, Head and Neck Surgery, University of Oldenburg, 26129 Oldenburg, Germany; 3 Institute of Human Genetics, Medical Faculty and University Hospital Düsseldorf, Heinrich Heine University Düsseldorf, 40225 Düsseldorf, Germany; 4 Research Center Neurosensory Science, University of Oldenburg, 26129 Oldenburg, Germany; 5 Institute of Medical Genetics, University Medicine Oldenburg, Carl von Ossietzky University, 26129 Oldenburg, Germany; 6 Cluster of Excellence “Hearing4All”, University of Oldenburg, 26129 Oldenburg, Germany

**Keywords:** hereditary hearing loss, genetic sequence variants, mutation, cochlear implant

## Abstract

**Background/Objectives:** Hearing loss is one of the most prevalent sensory disorders in humans, with genetic factors accounting for approximately 60% of cases. Cochlear implantation is an effective intervention for individuals with severe-to-profound hearing loss. However, substantial variability in postoperative auditory performance persists, complicating the prediction of individual outcomes. This study investigated the genetic findings associated with hearing loss in a cohort of seven affected adults with cochlear implants (CIs). **Methods:** A total of seven patients with severe-to-profound hearing loss underwent genetic testing. Two of them were part of diagnostic screening, and five of them were part of research genetic analyses. **Results:** High-throughput genomic DNA sequencing identified twelve variants across multiple genes, including four new sequence variants. Based on ACMG/AMP criteria, integrating computational predictions, population frequency data, ClinVar annotations, and in silico pathogenicity assessments, the identified variants were classified as pathogenic variants, likely pathogenic variants, and variants of uncertain significance (VUS). We detected one pathogenic variant, two likely pathogenic variants and nine variants of uncertain significance (VUS). Novel variants were further analyzed using multiple sequence alignment to assess evolutionary conservation. **Conclusions:** The identification of four novel variants within the analyzed patients underscores the genetic heterogeneity of hearing loss and the importance of genetic analyses for improving the understanding of its molecular basis. Further functional and clinical studies are required to determine the pathogenicity of these variants and their potential clinical relevance.

## 1. Introduction

Hearing loss is one of the most prevalent sensory disorders in humans. To date, more than 1.5 billion individuals worldwide are affected by this condition, with projections indicating that by 2050, every tenth individual will experience symptoms of hearing loss [1]. In northwestern Germany, the prevalence of hearing loss is approximately 16% [2]. This disorder significantly impairs quality of life, influencing communication and social interactions, and has been linked to increased risks of depression, hospitalization, and neurodegenerative diseases such as dementia [3]. The etiology of hearing loss is multifactorial, encompassing environmental factors, age-related degeneration, and genetic alterations [1].

Hearing loss can be classified into syndromic and nonsyndromic forms. Syndromic hearing loss is characterized by co-occurrence with other systemic anomalies, affecting multiple organ systems (e.g., Usher syndrome and Waardenburg syndrome), whereas nonsyndromic hearing loss is restricted to auditory dysfunctions. Nonsyndromic forms account for approximately 70% of genetic hearing loss cases [4,5]. To date, pathogenic variants in 156 genes have been reported to be associated with nonsyndromic hearing loss [6,7], although the strength of gene-disease evidence varies among these associations. These genes represent approximately 0.7% of all protein-coding genes in the human genome. The most frequently altered genes associated with hearing loss include *GJB2*, *MYO7A*, and *SLC26A4* [8,9,10,11,12]. In syndromic forms, it was estimated that several hundred genes are associated with hereditary hearing loss [7]. These genes encode proteins critical for the development, function, and maintenance of the auditory system, including ion channels, structural components, and intracellular signaling molecules [13].

The genetic heterogeneity of this clinical condition presents a significant challenge in identifying the molecular cause of the disease, managing clinical symptoms, and targeted treatment. Sequence variants in different genes can affect distinct components of the auditory pathway, resulting in various molecular alterations and clinical manifestations. Furthermore, the same genetic variant may exhibit variable expressivity, leading to differences in age of onset, severity, and clinical manifestations in affected individuals and/or members within the same family [13].

One treatment option for patients with severe-to-profound hearing loss is cochlear implantation. While the cochlear implant (CI) significantly improves auditory perception in many cases, approximately 3–7% of CI recipients do not experience benefit from the device [14,15]. Predicting CI outcomes preoperatively remains challenging. Multiple factors, including age at onset of hearing loss, duration of hearing loss, age at implantation, underlying etiology, hearing experience with CI, hearing aid use, CI brand, and percentage of active electrodes, have been identified as contributors to post-implantation performance [16,17,18,19,20]. In addition, emerging evidence indicates that genetic factors play a crucial role in determining CI outcomes by influencing auditory processing and speech perception [21,22,23,24]. The “spiral ganglion hypothesis” postulates that pathogenic variants in genes affecting neural structures are associated with reduced CI outcomes, whereas mutations in genes primarily affecting sensory cells are linked to more favorable post-implantation performance [21]. Further studies have also confirmed this hypothesis [23,24]. These findings highlighted the importance of genetic analyses for improving our understanding of the molecular basis of hearing loss and their potential contribution to personalized medical applications. Sequence variants can impact the sensory components of the auditory pathway, e.g., the organ of Corti and synaptic structures, as well as the neural partition, which in turn may influence speech perception following implantation [23]. Genetic analyses may contribute to the future optimization of CI strategies by supporting more individualized approaches to patient management.

In the present study, we performed genetic analyses of patients treated with CI to identify variants underlying severe-to-profound hearing loss. Variant interpretation was further performed by relating the sequence variant to clinical symptoms. The newly identified sequence variants were analyzed using multiple protein sequence alignments.

## 2. Materials and Methods

### 2.1. Patients

Seven patients with CI were clinically examined at the Otorhinolaryngology, Evangelical Hospital Oldenburg. Genetic analyses were conducted at the Human Genetics, University of Oldenburg, Oldenburg, Germany, and at the Medical Genetics, University Hospital, Oldenburg, Germany. The study adhered to the tenets of the Declaration of Helsinki. Informed consent was obtained from all subjects involved in the study. This study was registered and approved by the Medical Ethics Commission of the University of Oldenburg (2018-097).

### 2.2. Genomic DNA

Genomic DNA was extracted from peripheral blood samples collected in EDTA tubes. DNA isolation was performed according to the manufacturers’ instructions (GentraPuregene Kit, QIAGEN GmbH, Hilden, Germany, and Maxwell DNA-Extraction Systems, Promega, Walldorf, Germany). DNA concentration and purity were assessed using a NanoDrop spectrophotometer, and DNA integrity was evaluated by agarose electrophoresis.

### 2.3. High-Throughput Sequencing and Sanger Sequencing

Whole-exome sequencing (WES) was performed at the Human Genetics, University of Oldenburg, while whole-genome sequencing (WGS) was performed at the Medical Genetics, University Hospital, Oldenburg. Initial analyses focused on the *GJB2* and *GJB6* genes using either Sanger sequencing (patients 1, 3, 4, and 5) or sequencing data generated by the Illumina NovaSeq 6000 platform (Illumina, Inc., San Diego, CA, USA) (patients 2 and 6). The genetically unclarified patients were further analyzed using targeted NGS panels for known hearing loss-associated genes (details provided in Supplementary Materials). Two patients underwent diagnostic gene panel analyses (Table S1), and five patients were analyzed using a research in-house panel (Table S2). Tier 1 includes genes with definitive strong clinical validity for hearing loss, supported by substantial genetic and functional evidence establishing a clear gene-disease association. Tier 2 comprises genes with emerging or moderate evidence of association, for which clinical relevance is suggestive but not yet fully established.

### 2.4. Bioinformatic Analyses

WES and WGS data were analyzed using either the Varvis platform (VARVIS Version 1.15, Limbus Medical Technologies GmbH, Rostock, Germany) or the VarSeq platform (V.2.4.0; GoldenHelix Inc., Bozeman, MT, USA), respectively. Primary filtering criteria included prioritization of sequence variants with predicted high, moderate, or modifier impact on protein function. Allele frequency was set at a maximum of 1.5% for variants identified in the WES cohort (Human Genetics) and 1% for variants identified in the WGS cohort (Medical Genetics) based on the gnomAD database to initially remove population polymorphisms prior to further variant prioritization. Variants with increased frequency in local control cohorts or internal and external reference datasets were also excluded. Remaining variants were evaluated for functional relevance and inheritance mode. Variants classified as pathogenic, likely pathogenic or variants of uncertain significance (VUS) were further analyzed by correlating genotype with phenotype. Cases were considered genetically resolved after identification of sequence variants with sufficient evidence to support classification as pathogenic or likely pathogenic, in combination with a consistent clinical phenotype. Cases with VUS were considered genetically unresolved, although these variants were evaluated for potential phenotype consistency and may represent candidates for future functional validation.

### 2.5. Genetic Variant Classification

Variant filtering and interpretation were performed according to the common consensus recommendations of the American College of Medical Genetics and Genomics (ACMG) and relevant ClinGen specifications [25]. Allele frequencies were cross-referenced with gnomAD, and gene-specific inheritance patterns were obtained from the Online Mendelian Inheritance in Man (OMIM) database. For potentially interesting variants, variant annotation and interpretation were performed using Franklin software (https://franklin.genoox.com—Franklin by Genoox, accessed on 9 April 2026), an automated platform integrating multiple data sources including ClinVar, gnomAD, dbSNP, Human Gene Mutation Database (HGMD), and OMIM, to support ACMG/AMP-based variant interpretation. Suggested criteria from Franklin subsequently underwent manual validation integrating public databases (gnomAD, ClinVar, dbSNP, and Deafness Variation Database (DVD)), in silico prediction tools (CADD, REVEL and SpliceAI for assessment of potential splicing effects) and variant- and gene-specific publications.

### 2.6. Multiple Protein Sequence Alignments

Multiple sequence alignments (MSAs) for four novel variants were performed using Clustal Omega (version 1.2.4) [26] on orthologous proteins from human (*Homo sapiens*: *MYH9* (NP_002464.1), *PTPRQ* (NP_001138498.1), *PDE1C* (NP_001177986.1), *GATA3* (NP_001002295.1)), rat (*Rattus norvegicus*: *MYH9* (NP_001292806.1), *PTPRQ* (NP_075214.2), *PDE1C* (NP_112340.1), *GATA3* (NP_579827.1)), mouse (*Mus musculus*: *MYH9* (NP_071855.2), *PTPRQ* (NP_001357916.1), *PDE1C* (NP_001020739.1), *GATA3* (NP_001403975.1)), chicken (*Gallus gallus*: *MYH9* (NP_990808.2), *PTPRQ* (XP_040523460.1), *PDE1C* (XP_046768496.1), *GATA3* (NP_001008444.1)), fish (*Danio rerio*: *MYH9* (NP_001091647.3), *PTPRQ* (XP_073786317.1), *PDE1C* (XP_073786845.1), *GATA3* (NP_571286.1)), frog (*Xenopus laevis*: *MYH9* (NP_001081846.1), *PTPRQ* (XP_041442217.1), *PDE1C* (XP_041422066.1), *GATA3* (XP_018108227.1)), fly (*Drosophila melanogaster*: *MYH9* (NP_001246516.1), *PDE1C* (NP_001137816.1), *GATA3* (NP_731211.1)) and nematode (*Caernorhabditis elegans*: *MYH9* (NP_508504.2), *PDE1C* (NP_493343.1)).

## 3. Results

Herein, we present seven patients with hearing loss who were clinically and genetically analyzed. The majority of these patients (six) manifested nonsyndromic hearing loss, whereas one patient showed syndromic hearing loss. The cohort comprised five females and two male individuals, with a median age of 51 years old. All participants were unrelated (Table 1).

Genetic analyses revealed a total of twelve sequence variants in twelve genes associated with hearing loss (Table 2).

Variants were classified according to ACMG/AMP guidelines. In silico prediction tools, including CADD, REVEL, and SpliceAI, were used. Additionally, the Franklin platform was used for variant annotation and ACMG/AMP-based interpretation (Table 3).

### 3.1. Sequencing Analysis

**Figure 1:**
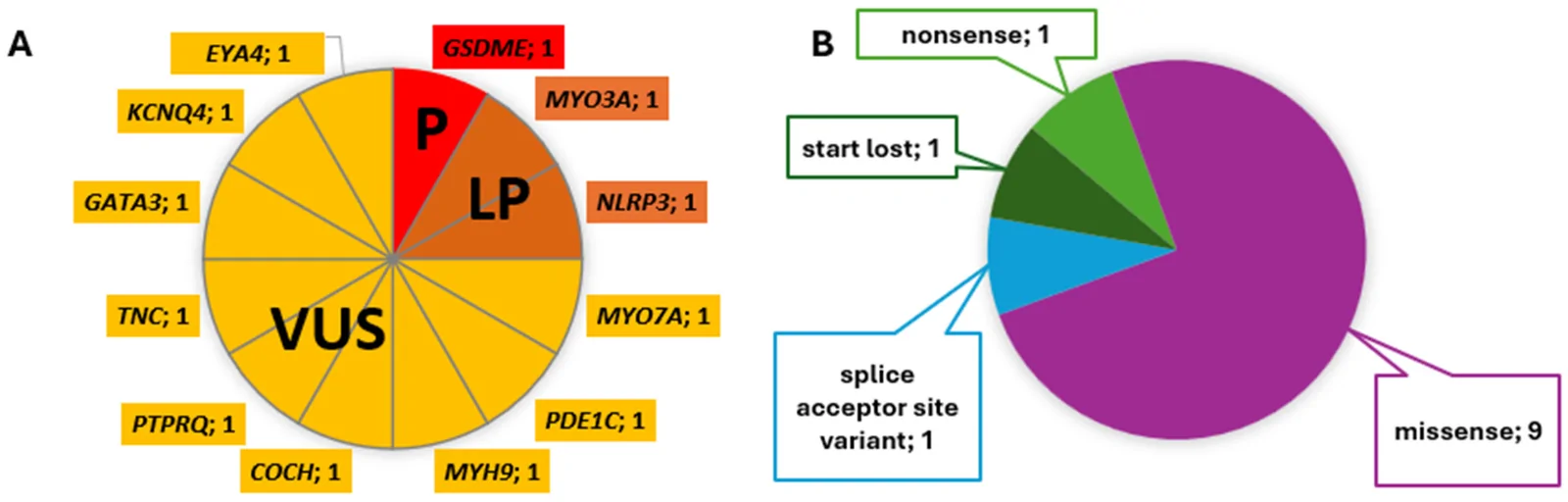
Altered genes and types of variants. (**A**): The detected variants were classified as pathogenic (red), likely pathogenic (orange) or VUS (yellow). The gene and the number of variants detected in the gene are shown. (**B**): The distribution of variant types and the total number of variants detected in this category. P = pathogenic; LP = likely pathogenic; VUS = variant of uncertain significance.

High-throughput sequencing identified single variants in twelve genes (*MYO3A*, *PTPRQ*, *GSDME*, *MYO7A*, *MYH9*, *NLRP3*, *PDE1C*, *COCH*, *TNC*, *GATA3*, *KCNQ4*, and *EYA4*). For the twelve detected variants, pathogenicity classifications from ACMG guidelines, Franklin, ClinVar, and DVD were collected, compared, and interpreted by the authors to derive an overall classification. One variant was classified as pathogenic, two as likely pathogenic, and nine as VUS (Table 1, Figure 1A). Missense variants constituted over 75% of the detected alterations. Additionally, a start-codon sequence variant, a noncanonical intronic splice-region variant, and a nonsense variant were identified (Table 1, Figure 1B).

### 3.2. Novel Variant Interpretation

We detected four novel genetic variants that were, to the best of our knowledge, not reported as being associated with hearing loss in public databases or the literature. Among these variants, all were classified as VUS (*PDE1C* (NM_001191057.4): c.1773_1774delCTinsTG (p.Ser592Ala); *MYH9* (NM_002473.5): c.5444A>C (p.Lys1815Thr); *PTPRQ* (NM_001145026.1): c.4016T>G (p.Val1339Gly); *GATA3* (NM_001002295.2): c.733A>C (p.Thr245Pro)).

**Figure 2:**
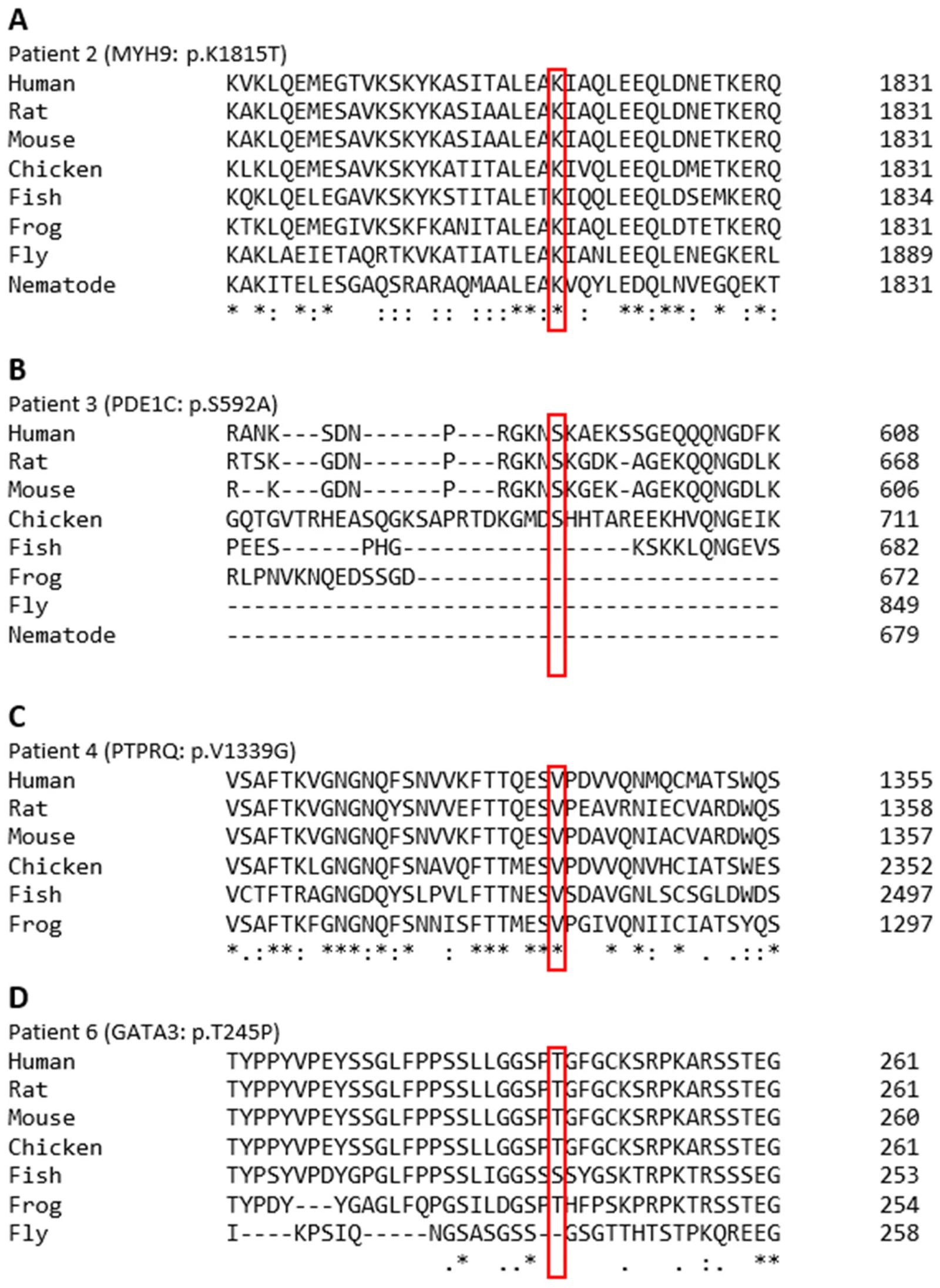
Multiple protein sequence alignments. The amino acid positions of the novel variants in MYH9 (**A**), PDE1C (**B**), PTPRQ (**C**) and GATA3 (**D**) across different species. The human wild-type amino acid residues and corresponding residues in orthologs are highlighted by red boxes. Conserved scores: * = identical residue; : = strongly similar; . = weakly similar; empty space = no similarity.

MSA revealed that variants in MYH9 (Figure 2A) and PTPRQ (Figure 2C) affect evolutionarily highly conserved amino acid residues across different species. PTPRQ lacked orthologs in fly and nematode. Notably, the variant in *PTPRQ* (NM_001145026.1): c.4016T>G (p.Val1339Gly) affected the 1st base pair of exon 25, a position that may influence transcript splicing. However, the effect of this alteration on splicing, transcript processing, and protein function cannot be reliably predicted without experimental validation. In silico splicing prediction tools (e.g., SpliceAI) did not indicate a predicted splicing effect (SpliceAI = 0). Therefore, this variant remains classified as a VUS. The sequence alteration in PDE1C changed an amino acid conserved among mammals and birds (Figure 2B), while the GATA3 variant showed broader conservation, extending to amphibians (Figure 2D).

Collectively, the amino acid substitutions detected herein may alter or destabilize regional protein structure, thereby compromising protein functions.

### 3.3. Genotype–Phenotype Correlations

Three patients carried a single variant in one gene (Table 1: Patient 2: *GSDME*; Patient 5: *TNC*; Patient 6: *GATA3*). While the *GSDME* variant is consistent with the clinical presentation, the contribution of *TNC* and *GATA3* variants remains uncertain, as both are classified as VUS. In four patients, variants in several genes were identified. However, interactions or multifactorial contributions to the phenotypic expression cannot be established based on genetic findings alone and require further functional and clinical investigation. Patient 3 presented variants in *MYO7A* and *MYH9* (Table 1), both associated with nonsyndromic hearing loss, concordant with the patient’s clinical presentation. Patient 4 exhibited variants in three genes: *NLRP3*, *PDE1C* and *COCH* (Table 1). This patient’s history of hearing loss, meningitis and postmeningitic ossification of the basal scala tympani of the right ear suggests an inflammatory etiology. Among these, the *NLRP3* variant, classified as likely pathogenic, is the most plausible disease-associated gene, given its known role in hearing loss, with a potentially inflammatory component (OMIM #617772).

Patient 7, who was diagnosed with Ménière’s disease—a disorder characterized by episodic vertigo, sensorineural hearing loss, tinnitus, and aural fullness [27,28]—carried variants in *KCNQ4* and *EYA4* (Table 1), both implicated in auditory dysfunction.

Among all patients, the phenotypic presentations encompassed both syndromic and nonsyndromic forms of hearing loss. In some patients, plausible genotype–phenotype correlations were detected (Table 1 and Table 2). In Patient 1, affected by syndromic hearing loss and diagnosed with arthrogryposis multiplex congenita (AMC), one variant in *MYO3A* and one variant in *PTPRQ* were identified (Table 1 and Table 2). However, both *MYO3A* and *PTPRQ* are linked to nonsyndromic forms of hearing loss (OMIM #606808, OMIM #603317), and thus the genetic basis of this patient’s syndromic phenotype remains unclear. Nevertheless, the variant in *MYO3A* was classified as likely pathogenic and represents a plausible contributor to the patient’s hearing loss. However, additional evidence is required to establish its causal role in this individual.

## 4. Discussion

This study provides a genetic analysis of seven patients with hearing loss who received CI. A total of twelve distinct genetic variants were identified. Nearly half of the patients harbored variants in known disease-associated genes, which were previously reported [29]. All identified variants were considered potentially consistent with patients’ clinical phenotypes, although the pathogenic contribution of the novel VUS remains uncertain.

Sequencing analyses revealed a previously reported variant in *GSDME* (NM_001127453.2): c.991-15_991-13delTTC (p.?). The sequence variants in *GSDME* associated with hearing loss have been shown to converge on a common molecular mechanism involving exon 8 skipping and have been identified both within the introns flanking exon 8 and within exon 8 itself [30,31,32]. Recent functional studies have demonstrated that exonic variants predicted to result in missense substitutions or frameshift alterations may instead exert their pathogenic effect by inducing aberrant exon 8 splicing, as confirmed by minigene assays. These findings highlight that the molecular consequences of *GSDME* variants cannot always be reliably inferred from genomic sequence alone and underscore the importance of functional assays for the interpretation of noncanonical and splice-region variants [32]. Additionally, a variant in *MYO3A* was identified. While *MYO3A* variants are typically implicated in nonsyndromic hearing loss, the concomitant diagnosis of AMC raises the possibility of an additional, unidentified genetic factor contributing to the syndromic phenotype. Given that the identified *MYO3A* variant is classified as likely pathogenic and is very likely responsible for the patient’s hearing loss, its contribution to the overall phenotype, particularly the coexistence of AMC, cannot currently be established. Alternatively, the *MYO3A* mutations may play a broader role than previously recognized, either contributing directly to the syndromic features or intensifying the severity of hearing loss in the context of a complex genetic syndrome. Nevertheless, further evidence is required to support this hypothesis.

Four patients carried multiple variants in different genes (patients 1, 3, 4 and 7), with some variants co-occurring alongside known disease-associated variants (patients 1, 3, 4). The genetic basis of hearing loss in complex cases, such as patients 4 and 7, remains challenging to elucidate. Patient 4 carried variants in *NLRP3*, *PDE1C*, and *COCH*, with the *NLRP3* variant being the most plausible cause of hearing loss, consistent with its known association with both inflammatory and non-inflammatory conditions (OMIM #617772). The patient’s history of meningitis and subsequent ossification of the basal scala tympani in the right ear may be consistent with prior reports associating *NLRP3* variants with both features [33,34,35]. To further evaluate a potential genotype–phenotype correlation, clinical re-evaluation, assessment of inflammatory markers, a detailed immunological history, FLAIR-MRI, and functional assays may be implicated. Functional characterization of NLRP3 variants may provide additional insights into their biological relevance, as increased inflammasome activation and IL-1β secretion have been reported for pathogenic *NLRP3* variants, potentially informing targeted therapeutic approaches [36]. The *PDE1C* variant and the *COCH* variant, both classified as VUS, may represent incidental findings or potential modifier variants. However, their contributory effect on hearing loss remains unclear.

Patient 6 carries a VUS in *GATA3* and is described as having nonsyndromic hearing loss. Because *GATA3* is associated with HDR (hypoparathyroidism, deafness, and renal disease) syndrome, interpreting the genotype–phenotype correlation in this patient requires careful clinical reassessment, segregation analysis, and, ideally, functional studies. However, neither hypoparathyroidism nor renal manifestations have been reported in this patient. A recent study demonstrated that pathogenic *GATA3* variants may present as apparently nonsyndromic hearing loss, highlighting the importance of comprehensive phenotypic evaluation and cautious interpretation of *GATA3* variants [37].

Patient 7, diagnosed with Ménière’s disease, exhibited variants in *KCNQ4* and *EYA4*. Although the genetic etiology of Ménière’s disease remains incompletely understood, potassium channel genes such as *KCNE1* and *KCNE3* have been implicated [38]. However, evidence remains inconclusive, potentially due to population-specific genetic differences [39,40]. Given that *KCNQ4* encodes a potassium channel, the identified variant may represent a potential candidate contributing to the patient’s hearing loss. However, gene function alone is insufficient to establish an allele-specific causal relationship. Nevertheless, neither *KCNQ4* nor *EYA4* has been established as a causative gene for Ménière’s disease. Current evidence suggests that Ménière’s disease is a multifactorial disorder with an incompletely understood etiology. Multiple factors, including genetic susceptibility, immune dysregulation, viral infections, and trauma, have been proposed to contribute to disease development, although the relative contribution of each remains uncertain [41]. Therefore, although *KCNQ4* and *EYA4* are established genes associated with autosomal dominant hearing loss, the identified variants are classified as VUS and should not be interpreted as the genetic cause of the patient’s hearing loss or clinical diagnosis of Ménière’s disease. Accordingly, comprehensive phenotypic characterization, including the diagnostic criteria (recurrent spontaneous vertigo episodes, documented sensorineural hearing loss, fluctuating aural symptoms, and exclusion of alternative vestibular diagnoses) used, vertigo history, tinnitus, aural fullness, fluctuating hearing loss, and imaging findings, is essential when interpreting potential genetic associations in patients with Ménière’s disease [41,42].

These findings raise the possibility that multiple genetic variants may contribute to phenotypic variability through mechanisms such as modifier effects, additive or digenic contributions, or polygenic inheritance. In addition, phenotypic outcomes might be influenced by environmental factors or epigenetic mechanisms [43]. However, these hypotheses require validation in larger cohorts and functional studies.

The identification of four previously unreported genetic variants, all classified as VUS (*PDE1C* (NM_001191057.4): c.1773_1774delCTinsTG (p.Ser592Ala), *MYH9* (NM_002473.5): c.5444A>C (p.Lys1815Thr), *PTPRQ* (NM_001145026.1): c.4016T>G (p.Val1339Gly), *GATA3* (NM_001002295.2): c.733A>C (p.Thr245Pro)), expands the spectrum of reported variants in hearing loss-associated genes. However, their clinical significance remains uncertain pending additional genetic and functional evidence. Notably, two variants reported at the same *PDE1C* transcript position (*PDE1C* (NM_001191057.4): c.1773C>T (p.Asn591=); *PDE1C* (NM_001191057.4): c.1774T>G (p.Ser592Ala)) have been previously classified as benign or likely benign by two independent sources. The presence of a ClinVar-reported pathogenic variant affecting the same genomic position as the identified *GATA3* variant further highlights the potential relevance of these genetic loci. However, the clinical significance of the identified variant remains uncertain and would require additional evidence, e.g., by verifying segregation of the variant with the disease.

Pathogenic and likely pathogenic variants frequently locate to evolutionarily conserved gene regions, presumably consistent with their functional relevance. MSA revealed that variants in *MYH9* and *PTPRQ* reside within highly conserved regions across diverse taxa, which may indicate evolutionary constraint of these protein regions. In contrast, variants located in less conserved protein regions may be less likely to disrupt protein function. However, evolutionary conservation alone is not sufficient for variant pathogenicity.

Several variants are located within functionally critical domains, including the von Willebrand factor (vWF) type A domain of COCH, which is involved in extracellular matrix formation [44]. Previously reported pathogenic variants within this domain have been shown to promote protein aggregation rather than dimerization [45]. Whether the variant identified in the present study has a similar effect remains unknown. The *MYH9* variant resides in the myosin tail domain. If functionally relevant, structural alterations in this domain could impair cytoskeletal integrity of hair cells and stereocilia maintenance [46]. The *PDE1C* gene is expressed in the cytosol of both inner and outer hair cells and is involved in cyclic nucleotide signaling and lysosomal processes, supporting a potential role in Ca^2+^ homeostasis [47,48]. *GATA3* is a transcription factor that regulates inner ear formation, particularly hair cell differentiation and maturation, as well as spiral ganglion neuron (SGN) development and maintenance [49,50,51]. Similarly, *PTPRQ* encodes the protein tyrosine phosphatase receptor Q, a protein critical for hair cell and stereocilia development during the late embryonic and early postnatal stages [52].

The identification of novel variants in more than half of the cases highlights the potential of genetic testing to expand the spectrum of variants associated with hearing loss. However, because all novel variants were classified as VUS, their contribution to the observed phenotypes remains uncertain and should be interpreted cautiously. Importantly, additional undetected sequence variants and modifier variants might also contribute to the variable clinical phenotype of the patients. To elucidate the clinical relevance of these variants, functional assays and larger patient cohorts would be required. Robust analyses, ideally conducted within international multicenter consortia and incorporating co-segregation studies, are necessary to determine the variant pathogenicity and to better explain possible contributions of genetic defects to auditory impairment.

Given that all genes identified in this study are expressed in the sensory compartment of the cochlea, including hair cells and the cochlear duct, it may be hypothesized that patients harboring pathogenic variants in these genes could benefit from cochlear implantation because the device bypasses the sensory apparatus. However, several genes, including *GSDME*, *NLRP3*, *GATA3* and *EYA4*, exhibit additional expression in SGN, suggesting that differences in the extent of neural involvement may contribute to variability in CI outcomes [21,23]. Thus, patients carrying variants in these genes may exhibit heterogeneous post-implantation auditory performance, with potentially limited benefit. However, this association requires further clinical and functional investigation.

While the impact of *GSDME* variants on CI performance remains poorly characterized, recent studies reported patients with long-term hearing loss carrying the same variant (*GSDME* (NM_001127453.2): c.991-15_991-13delTTC (p.?)), with variable outcomes, ranging from absent auditory perception to transient improvements followed by deterioration [53,54]. *GSDME* is highly expressed in cochlear hair cells, and its dysfunction leads to auditory impairment [55], potentially mediated through apoptosis of these cells [56]. Furthermore, degeneration of the stria vascularis and spiral ligament was reported from histopathology [54]. Given the *GSDME* expression also in the SGN, it is hypothesized that patients may only poorly benefit from CI provision.

In contrast, patients carrying *NLRP3* variants have demonstrated CI outcomes ranging from 60% to 100% in various speech perception tests at 12-month follow-up [35]. Given the involvement of NLRP3 in cochlear and SGN function [35,57,58], CI success in these cases may be influenced by factors beyond genetic predisposition, complicating the prediction of individual patient outcomes. Notably, NLRP3 and GSDME are involved in the same inflammatory pathway [59,60].

To the best of our knowledge, the influence of the *GATA3* variants has not been characterized on CI outcomes in speech discrimination. Prior studies reported absence of distortion product otoacoustic emissions (DPOAEs) alongside preserved auditory brainstem responses (ABRs), indicating outer hair cell (OHC) dysfunction with intact neural transmission [50]. Similarly, progressive OHC degeneration, later also affecting all hair cells, and a 30 dB hearing loss in ABRs have been reported [49]. These data suggest that pathogenic *GATA3* variants primarily affect cochlear sensory structures, indicating potential benefit from cochlear implantation.

*EYA4* is a transcriptional regulator primarily expressed in the organ of Corti, including hair cells and supporting cells, and also in spiral ganglion nuclei. It plays an essential role in maintaining cochlear structural integrity [61]. Consequently, pathogenic *EYA4* variants may affect both the sensory and neural partitions, although CI outcomes remain poorly described. Tropitzsch and colleagues [24] categorized *EYA4* as a neural gene and reported a CI outcome of 55%, supporting potential benefit from implantation [24].

While genetic factors have increasingly been recognized as potential determinants of CI performance, their influence remains insufficiently understood [23,29,62,63]. Cochlear implants remain the standard treatment for severe-to-profound hearing loss, but are associated with high outcome variability, difficulties in spatial hearing, and differences in hearing from natural auditory perception. As the CI is primarily used to manage the symptoms, gene therapy may potentially cure and restore hearing. Recent success has been demonstrated in *OTOF*-associated hearing loss, where patients treated by gene therapy regained near-physiological hearing, marking the first effective gene therapy for hereditary hearing loss in clinical trials [64,65,66].

Numerous studies have developed gene therapy approaches in animal models for several genes. Experimental approaches including adenovirus-, lentivirus- and CRISPR-based therapies, as well as antisense oligonucleotides, have shown variable success in animal models for genes such as *MYO7A* [67,68,69], *COCH* [70], and *KCNQ4* [71,72,73]. A bioRxiv preprint additionally reported a CRISPR-based approach in an *MYO7A* model [69].

Nevertheless, a number of hearing loss-associated genes, including *GSDME*, *MYH9*, *NLRP3*, *GATA3*, *PDE1C*, *MYO3A*, *PTPRQ*, *TNC*, and *EYA4*, have not yet been addressed by gene therapy. Currently, management of these conditions remains largely symptomatic, relying on hearing aids or cochlear implantation, with some pharmacological strategies under investigation, particularly for *NLRP3*-associated inflammation [36,74,75,76].

Despite its potential, gene therapy faces several challenges, including the selection of appropriate therapeutic strategies [77], optimization of delivery vectors, identification of effective delivery routes [78], and uncertainty regarding long-term efficacy [79]. Similar to CI, outcomes may remain variable [78] and be influenced by disease mechanisms and timing of intervention. Therefore, understanding disease mechanisms is crucial for developing personalized treatment options.

This study has limitations. Firstly, the small cohort size does not allow for general conclusions. Secondly, two patients initially underwent diagnostic gene panel analyses as part of routine clinical care. They subsequently provided informed consent for the use of their genetic data in this research study. In contrast, five patients were analyzed directly using a research in-house panel. The research panel comprised a total of 386 genes, encompassing all 252 genes included in the diagnostic panel and supplemented with additional genes previously implicated in hearing loss. This expanded design provided broader coverage of both established and emerging hearing loss-associated genes. Thirdly, another limitation is that genetic segregation data were unavailable. Therefore, the pathogenic contribution of the novel variants remains speculative. Regarding novel variants, this study relies primarily on evolutionary conservation, protein alignments, and database searches. To establish the pathogenicity of novel variants, functional assays are needed. Of note, additional genetic, epigenetic, or environmental factors contributing to the phenotype cannot be excluded.

## 5. Conclusions

In conclusion, this study represents a descriptive case series that expands the spectrum of genetic variants identified in patients with hearing loss undergoing cochlear implantation by reporting both known and novel variants in genes associated with syndromic and nonsyndromic hearing loss. Although the small cohort precludes definitive conclusions regarding genotype–phenotype correlations or associations with cochlear implant outcomes, these findings provide additional evidence supporting the genetic heterogeneity of hearing loss and generate hypotheses for future studies. Further investigations in larger, well-characterized cohorts, together with functional analyses of variants of uncertain significance, are required to clarify their clinical relevance and potential contribution to variability in cochlear implant outcomes.

## Figures and Tables

**Table 1:** Clinical characteristics.

Patient	Clinical Diagnosis	Age of Diagnosis (Years)	Sex	Ancestry/Ethnicity
1	Syndromic hearing loss (Arthrogryposis multiplex congenita (AMC))	33	f	Lebanon
2	Nonsyndromic hearing loss	76	f	unknown
3	Nonsyndromic hearing loss	50	f	Germany
4	Nonsyndromic hearing loss, meningitis with postmeningitic ossification of the basal scala tympani in the right ear	30	f	Germany
5	Nonsyndromic hearing loss	75.5	f	Germany
6	Nonsyndromic hearing loss	51	m	unknown
7	Nonsyndromic hearing loss (Ménière’s disease)	58	m	unknown

m = masculine, f = feminine.

**Table 2:** Sequencing findings.

Patient	Applied Panel	Sequencing Method	Gene	NM_Number (MANE)	Nucleotide Change	Amino Acid Change	Molecular Consequence	Zygosity	Likely New Variant, According to ClinVar, PubMed Search, or GnomAD (All Databases Accessed on 9 April 2026)
1	Research_panel	Sanger Sequencing, NGS (WES)	*MYO3A*	NM_017433.5	c.76_77delinsTA	p.Thr26Ter	nonsense	het.	
			*PTPRQ*	NM_001145026.1	c.4016T>G	p.Val1339Gly	missense	het.	novel
2	Diagnostic_panel (Tier 1, Tier 2)	NGS (WGS)	*GSDME*	NM_001127453.2	c.991-15_991-13delTTC	p.?	noncanonical intronic splice-region	het.	
3	Research_panel	Sanger Sequencing, NGS (WES)	*MYO7A*	NM_000260.4	c.1373A>G	p.Asn458Ser	missense	het.	
			*MYH9*	NM_002473.5	c.5444A>C	p.Lys1815Thr	missense	het.	novel
4	Research_panel	Sanger Sequencing, NGS (WES)	*NLRP3*	NM_001243133.2	c.2575T>C	p.Tyr859His	missense	het.	
			*PDE1C*	NM_001191057.4	c.1773_1774delCTinsTG	p.Ser592Ala	missense	het.	novel
			*COCH*	NM_004086.3	c.692C>T	p.Ala231Val	missense	het.	
5	Research_panel	Sanger Sequencing, NGS (WES)	*TNC*	NM_002160.4	c.6494A>G	p.Gln2165Arg	missense	het.	
6	Diagnostic_panel (Tier 1, Tier 2)	NGS (WGS)	*GATA3*	NM_001002295.2	c.733A>C	p.Thr245Pro	missense	het.	novel
7	Research_panel	Sanger Sequencing, NGS (WES)	*KCNQ4*	NM_004700.4	c.3G>T	p.Met1?	start lost	het.	
			*EYA4*	NM_004100.5	c.1035G>C	p.Arg345Ser	missense	het.	

Genetic findings are ordered by clinical relevance. For patients with multiple variants, the most likely disease-causing gene is listed first, followed by additional variants in decreasing order of pathogenicity. Tier 1 indicates genes with strong, definitive evidence for a causal association with hearing loss; Tier 2 indicates genes with moderate or emerging evidence, suggesting an association but lacking definitive clinical validation. het. = heterozygous; ? = the effect of the variant on the protein level cannot be predicted.

**Table 3:** Variant interpretation.

Patient	Gene	Allele Frequency (GnomAD)	Inheritance (OMIM)	ACMG Criteria	CADD	REVEL	SpliceAI	dbSNP ID	ClinVar ID	DVD	Franklin (Genoox)	Authors’ Interpretation
1	*MYO3A*	not found	AR, AD	PVS1, PM2_sup	-	-	0	rs2491707628	1878816; 1 × P/1 × LP	LP	P	Likely pathogenic
	*PTPRQ*	not found	AR, AD	PM2_sup	-	-	0	-	-	-	VUS	VUS
2	*GSDME*	3.73 × 10^−6^	AD	PS4, PS3, PP1_str, PM2_sup	-	-	0.02	rs727505273	179997; 14 × P	P	LP	Pathogenic
3	*MYO7A*	1.50 × 10^−5^	AR, AD	PM2_sup, PM5, PP3	23.5	0.867	0.01	rs121965084	3627985; 2 × VUS	VUS	LP	VUS
	*MYH9*	not found	AD	PM2_sup, PP3, PP2	-	.	0	-	-	-	VUS	VUS
4	*NLRP3*	not found	AD	PS4, PM5, PM2_sup, PP2	21.4	0.296	0.02	rs2103174031	1188124; 1 × P/4 × LP	LP	LP	Likely pathogenic
	*PDE1C*	not found	AD	PM2_sup	-	-	0	-	-	-	VUS	VUS
	*COCH*	1.16 × 10^−4^	AR, AD	PP3	28.7	0.806	0.14	rs536511561	4538054; 1 × VUS	VUS	VUS	VUS
5	*TNC*	1.08 × 10^−4^	AD	PM2_sup, PP3	28.6	0.536	0.03	rs200330029	637026; 2 × VUS	VUS	VUS	VUS
6	*GATA3*	not found	AD	PM2_sup	21.8	0.439	0.01	rs1241118830	-	VUS	VUS	VUS
7	*KCNQ4*	2.21 × 10^−6^	AD	PVS1_mod, PM2_sup	22.2	-	0	-	-	VUS	VUS	VUS
	*EYA4*	6.20 × 10^−4^	AD	no criteria applicable	20.9	0.578	0.01	rs140170914	192118; 3 × VUS/5 × LB	VUS	VUS	VUS

DVD = Deafness Variation Database; AR = autosomal recessive, AD = autosomal dominant; P = pathogenic, LP = likely pathogenic, VUS = variant of uncertain significance, LB = likely benign.

## Data Availability

The data presented in this study are available upon reasonable request from the corresponding author.

## Supplementary Materials

The following supporting information can be downloaded at https://www.mdpi.com/article/10.3390/genes17080942/s1: Table S1: Tier 1 & 2; Table S2: Research panel.

## Abbreviations

The following abbreviations are used in this manuscript:

CI	Cochlear implant
WES	Whole-exome sequencing
WGS	Whole-genome sequencing
VUS	Variant of uncertain significance
OMIM	Online Mendelian Inheritance in Man
ACMG	American College of Medical Genetics and Genomics
MSA	Multiple sequence alignment
AMC	Arthrogryposis multiplex congenita
vWF	von Willebrand factor
SGN	Spiral ganglion neuron
DPOAEs	Distortion product otoacoustic emissions
ABRs	Auditory brainstem responses
OHC	Outer hair cell
