# Supplementary material for: Genetic Findings in Seven Cochlear Implanted Patients with Severe-to-Profound Hearing Loss
Source: Genes (Basel). 2026 Aug 12;17(8):942. doi: 10.3390/genes17080942 (PMC13511975; doi:10.3390/genes17080942)
Supplement: Supplementary file 1 [file genes-17-00942-s001.zip › genes-4389218-supplementary.pdf]

Supplements

Panels used for NGS. Tier 1 and 2 refer to genes with strong correlation with hearing loss and genes with moderate or emerging evidence suggesting an association but lacking full clinical validation, respectively.

Table S1. Tier 1 & 2

Gene name	Ref. Genome	Ensemble no.
ACTG1	GRCh38/ hg38	ENSG00000184009
ADGRV1	GRCh38/ hg38	ENSG00000164199
ATP1A3	GRCh38/ hg38	ENSG00000105409
ATP6V1B2	GRCh38/ hg38	ENSG00000147416
BMP4	GRCh38/ hg38	ENSG00000125378
CDH23	GRCh38/ hg38	ENSG00000107736
CEACAM16	GRCh38/ hg38	ENSG00000213892
CHD7	GRCh38/ hg38	ENSG00000171316
COCH	GRCh38/ hg38	ENSG00000100473
COL11A1	GRCh38/ hg38	ENSG00000060718
COL11A2	GRCh38/ hg38	ENSG00000204248
COL2A1	GRCh38/ hg38	ENSG00000139219
COL9A1	GRCh38/ hg38	ENSG00000112280
DIAPH1	GRCh38/ hg38	ENSG00000131504
DNMT1	GRCh38/ hg38	ENSG00000130816
DSPP	GRCh38/ hg38	ENSG00000152591
EDN3	GRCh38/ hg38	ENSG00000124205
EDNRA	GRCh38/ hg38	ENSG00000151617
EDNRB	GRCh38/ hg38	ENSG00000136160
EFTUD2	GRCh38/ hg38	ENSG00000108883
EPS8L2	GRCh38/ hg38	ENSG00000177106
EYA1	GRCh38/ hg38	ENSG00000104313
EYA4	GRCh38/ hg38	ENSG00000112319
FGF10	GRCh38/ hg38	ENSG00000070193
FGFR2	GRCh38/ hg38	ENSG00000066468
FOXI3	GRCh38/ hg38	ENSG00000214336
GATA3	GRCh38/ hg38	ENSG00000107485
GJB2	GRCh38/ hg38	ENSG00000165474
GJB3	GRCh38/ hg38	ENSG00000188910
GJB6	GRCh38/ hg38	ENSG00000121742
GNAI3	GRCh38/ hg38	ENSG00000065135
GREB1L	GRCh38/ hg38	ENSG00000141449
GRHL2	GRCh38/ hg38	ENSG00000083307
GSDME	GRCh38/ hg38	ENSG00000105928
HOXA2	GRCh38/ hg38	ENSG00000105996
HSPA9	GRCh38/ hg38	ENSG00000113013
KCNE1	GRCh38/ hg38	ENSG00000180509
KCNQ1	GRCh38/ hg38	ENSG00000053918
KCNQ4	GRCh38/ hg38	ENSG00000117013

KIT	GRCh38/ hg38	ENSG00000157404
KMT2D	GRCh38/ hg38	ENSG00000167548
LMX1A	GRCh38/ hg38	ENSG00000162761
MITF	GRCh38/ hg38	ENSG00000187098
MN1	GRCh38/ hg38	ENSG00000169184
MYH14	GRCh38/ hg38	ENSG00000105357
MYH9	GRCh38/ hg38	ENSG00000100345
MYO6	GRCh38/ hg38	ENSG00000196586
MYO7A	GRCh38/ hg38	ENSG00000137474
OPA1	GRCh38/ hg38	ENSG00000198836
ORC4	GRCh38/ hg38	ENSG00000115947
OSBPL2	GRCh38/ hg38	ENSG00000130703
OTX2	GRCh38/ hg38	ENSG00000165588
P2RX2	GRCh38/ hg38	ENSG00000187848
PAX2	GRCh38/ hg38	ENSG00000075891
PAX3	GRCh38/ hg38	ENSG00000135903
PBX1	GRCh38/ hg38	ENSG00000185630
PDZD7	GRCh38/ hg38	ENSG00000186862
PLCB4	GRCh38/ hg38	ENSG00000101333
PLS1	GRCh38/ hg38	ENSG00000120756
PNPT1	GRCh38/ hg38	ENSG00000138035
POLR1A	GRCh38/ hg38	ENSG00000068654
POLR1D	GRCh38/ hg38	ENSG00000186184
POU4F3	GRCh38/ hg38	ENSG00000091010
PTPRQ	GRCh38/ hg38	ENSG00000139304
SALL1	GRCh38/ hg38	ENSG00000103449
SALL4	GRCh38/ hg38	ENSG00000101115
SF3B4	GRCh38/ hg38	ENSG00000143368
SIX1	GRCh38/ hg38	ENSG00000126778
SLC12A2	GRCh38/ hg38	ENSG00000064651
SLC17A1	GRCh38/ hg38	ENSG00000124568
SOX10	GRCh38/ hg38	ENSG00000100146
SOX2	GRCh38/ hg38	ENSG00000181449
TBC1D24	GRCh38/ hg38	ENSG00000162065
TCOF1	GRCh38/ hg38	ENSG00000070814
TECTA	GRCh38/ hg38	ENSG00000109927
TFAP2A	GRCh38/ hg38	ENSG00000137203
TMC1	GRCh38/ hg38	ENSG00000165091
USP48	GRCh38/ hg38	ENSG00000090686
WFS1	GRCh38/ hg38	ENSG00000109501
AIFM1	GRCh38/ hg38	ENSG00000156709
COL4A5	GRCh38/ hg38	ENSG00000188153
KDM6A	GRCh38/ hg38	ENSG00000147050
OFD1	GRCh38/ hg38	ENSG00000046651
POU3F4	GRCh38/ hg38	ENSG00000196767
PRPS1	GRCh38/ hg38	ENSG00000147224
SMPX	GRCh38/ hg38	ENSG00000091482

TIMM8A	GRCh38/ hg38	ENSG00000126953
ABHD12	GRCh38/ hg38	ENSG00000100997
ALMS1	GRCh38/ hg38	ENSG00000116127
AP1S1	GRCh38/ hg38	ENSG00000106367
ATP6V1B1	GRCh38/ hg38	ENSG00000116039
BCS1L	GRCh38/ hg38	ENSG00000074582
BSND	GRCh38/ hg38	ENSG00000162399
CABP2	GRCh38/ hg38	ENSG00000167791
CDC14A	GRCh38/ hg38	ENSG00000079335
CDC6	GRCh38/ hg38	ENSG00000094804
CDT1	GRCh38/ hg38	ENSG00000167513
CEP250	GRCh38/ hg38	ENSG00000126001
CEP78	GRCh38/ hg38	ENSG00000148019
CIB2	GRCh38/ hg38	ENSG00000136425
CISD2	GRCh38/ hg38	ENSG00000145354
CLDN14	GRCh38/ hg38	ENSG00000159261
CLDN9	GRCh38/ hg38	ENSG00000213937
CLPP	GRCh38/ hg38	ENSG00000125656
CLRN1	GRCh38/ hg38	ENSG00000163646
DHODH	GRCh38/ hg38	ENSG00000102967
EIF4A3	GRCh38/ hg38	ENSG00000141543
EPS8	GRCh38/ hg38	ENSG00000151491
ESPN	GRCh38/ hg38	ENSG00000187017
ESRRB	GRCh38/ hg38	ENSG00000119715
FDXR	GRCh38/ hg38	ENSG00000161513
FGF3	GRCh38/ hg38	ENSG00000186895
FOXI1	GRCh38/ hg38	ENSG00000168269
FRAS1	GRCh38/ hg38	ENSG00000138759
FREM2	GRCh38/ hg38	ENSG00000150893
GGPS1	GRCh38/ hg38	ENSG00000152904
GIPC3	GRCh38/ hg38	ENSG00000179855
GPSM2	GRCh38/ hg38	ENSG00000121957
GRIP1	GRCh38/ hg38	ENSG00000155974
GRXCR1	GRCh38/ hg38	ENSG00000215203
GSC	GRCh38/ hg38	ENSG00000133937
HAAO	GRCh38/ hg38	ENSG00000162882
HMX1	GRCh38/ hg38	ENSG00000215612
HSD17B4	GRCh38/ hg38	ENSG00000133835
ILDR1	GRCh38/ hg38	ENSG00000145103
KARS1	GRCh38/ hg38	ENSG00000065427
KCNJ10	GRCh38/ hg38	ENSG00000177807
KCNJ16	GRCh38/ hg38	ENSG00000153822
LARS2	GRCh38/ hg38	ENSG00000011376
LHFPL5	GRCh38/ hg38	ENSG00000197753
LOXHD1	GRCh38/ hg38	ENSG00000167210
LRTOMT	GRCh38/ hg38	ENSG00000284922
MARVELD2	GRCh38/ hg38	ENSG00000152939

MASP1	GRCh38/ hg38	ENSG00000127241
MPZL2	GRCh38/ hg38	ENSG00000149573
MSRB3	GRCh38/ hg38	ENSG00000174099
MYO15A	GRCh38/ hg38	ENSG00000091536
MYO3A	GRCh38/ hg38	ENSG00000095777
OGDHL	GRCh38/ hg38	ENSG00000197444
ORC1	GRCh38/ hg38	ENSG00000085840
ORC6	GRCh38/ hg38	ENSG00000091651
OTOA	GRCh38/ hg38	ENSG00000155719
OTOF	GRCh38/ hg38	ENSG00000115155
OTOG	GRCh38/ hg38	ENSG00000188162
OTOGL	GRCh38/ hg38	ENSG00000165899
PCDH15	GRCh38/ hg38	ENSG00000150275
PJVK	GRCh38/ hg38	ENSG00000204311
POLR1C	GRCh38/ hg38	ENSG00000171453
RDX	GRCh38/ hg38	ENSG00000137710
RNF220	GRCh38/ hg38	ENSG00000187147
S1PR2	GRCh38/ hg38	ENSG00000267534
SERAC1	GRCh38/ hg38	ENSG00000122335
SERPINB6	GRCh38/ hg38	ENSG00000124570
SGPL1	GRCh38/ hg38	ENSG00000166224
SLC26A4	GRCh38/ hg38	ENSG00000091137
SLC4A11	GRCh38/ hg38	ENSG00000088836
SLC52A2	GRCh38/ hg38	ENSG00000185803
SLC52A3	GRCh38/ hg38	ENSG00000101276
SLITRK6	GRCh38/ hg38	ENSG00000184564
SPATA5	GRCh38/ hg38	ENSG00000145375
SPATA5L1	GRCh38/ hg38	ENSG00000171763
STRC	GRCh38/ hg38	ENSG00000242866
SYNE4	GRCh38/ hg38	ENSG00000181392
TMIE	GRCh38/ hg38	ENSG00000181585
TMPRSS3	GRCh38/ hg38	ENSG00000160183
TRPN	GRCh38/ hg38	ENSG00000176058
TRIOBP	GRCh38/ hg38	ENSG00000100106
USH1C	GRCh38/ hg38	ENSG00000006611
USH1G	GRCh38/ hg38	ENSG00000182040
USH2A	GRCh38/ hg38	ENSG00000042781
WHRN	GRCh38/ hg38	ENSG00000095397
ABCC1	GRCh38/ hg38	ENSG00000124574
ACOX1	GRCh38/ hg38	ENSG00000161533
ANKH	GRCh38/ hg38	ENSG00000154122
ARSG	GRCh38/ hg38	ENSG00000141337
ATP11A	GRCh38/ hg38	ENSG00000068650
ATP2B2	GRCh38/ hg38	ENSG00000157087
CACNA1D	GRCh38/ hg38	ENSG00000157388
CCDC50	GRCh38/ hg38	ENSG00000152492
CDKN1C	GRCh38/ hg38	ENSG00000129757

COG4	GRCh38/ hg38	ENSG00000103051
COL9A2	GRCh38/ hg38	ENSG00000049089
COL9A3	GRCh38/ hg38	ENSG00000092758
CRYM	GRCh38/ hg38	ENSG00000103316
DIABLO	GRCh38/ hg38	ENSG00000184047
DIAPH3	GRCh38/ hg38	ENSG00000139734
DMXL2	GRCh38/ hg38	ENSG00000104093
ELMOD3	GRCh38/ hg38	ENSG00000115459
GDF6	GRCh38/ hg38	ENSG00000156466
HOMER2	GRCh38/ hg38	ENSG00000103942
KDM3B	GRCh38/ hg38	ENSG00000120733
KITLG	GRCh38/ hg38	ENSG00000049130
MCM2	GRCh38/ hg38	ENSG00000073111
MET	GRCh38/ hg38	ENSG00000105976
MORC2	GRCh38/ hg38	ENSG00000133422
PEX6	GRCh38/ hg38	ENSG00000124587
PMP22	GRCh38/ hg38	ENSG00000109099
RIPOR2	GRCh38/ hg38	ENSG00000111913
RPS28	GRCh38/ hg38	ENSG00000233927
SLC44A4	GRCh38/ hg38	ENSG00000204385
THOC1	GRCh38/ hg38	ENSG00000079134
TNC	GRCh38/ hg38	ENSG00000041982
TOP2B	GRCh38/ hg38	ENSG00000077097
TUBB4B	GRCh38/ hg38	ENSG00000188229
TWNK	GRCh38/ hg38	ENSG00000107815
YARS1	GRCh38/ hg38	ENSG00000134684
COL4A6	GRCh38/ hg38	ENSG00000197565
CEMIP	GRCh38/ hg38	ENSG00000103888
FOXF2	GRCh38/ hg38	ENSG00000137273
PSIP1	GRCh38/ hg38	ENSG00000164985
SGO2	GRCh38/ hg38	ENSG00000163535
SIX5	GRCh38/ hg38	ENSG00000177045
SNAI2	GRCh38/ hg38	ENSG00000019549
SPATC1L	GRCh38/ hg38	ENSG00000160284
STX4	GRCh38/ hg38	ENSG00000103496
STXBP3	GRCh38/ hg38	ENSG00000116266
TMTC2	GRCh38/ hg38	ENSG00000179104
BTD	GRCh38/ hg38	ENSG00000169814
CLIC5	GRCh38/ hg38	ENSG00000112782
CLRN2	GRCh38/ hg38	ENSG00000249581
CRLS1	GRCh38/ hg38	ENSG00000088766
DCDC2	GRCh38/ hg38	ENSG00000146038
DNAJC3	GRCh38/ hg38	ENSG00000102580
ESRP1	GRCh38/ hg38	ENSG00000104413
EXOSC2	GRCh38/ hg38	ENSG00000130713
GRXCR2	GRCh38/ hg38	ENSG00000204928
HARS2	GRCh38/ hg38	ENSG00000112855

HGF	GRCh38/ hg38	ENSG00000019991
IARS2	GRCh38/ hg38	ENSG00000067704
LETM1	GRCh38/ hg38	ENSG00000168924
MAN2B1	GRCh38/ hg38	ENSG00000104774
MANBA	GRCh38/ hg38	ENSG00000109323
NARS2	GRCh38/ hg38	ENSG00000137513
OXR1	GRCh38/ hg38	ENSG00000164830
PDSS1	GRCh38/ hg38	ENSG00000148459
PEX1	GRCh38/ hg38	ENSG00000127980
PEX26	GRCh38/ hg38	ENSG00000215193
PHYH	GRCh38/ hg38	ENSG00000107537
PPIP5K2	GRCh38/ hg38	ENSG00000145725
ROR1	GRCh38/ hg38	ENSG00000185483
SLC26A5	GRCh38/ hg38	ENSG00000170615
SPNS2	GRCh38/ hg38	ENSG00000183018
SPTBN4	GRCh38/ hg38	ENSG00000160460
TJP2	GRCh38/ hg38	ENSG00000119139
TMEM132E	GRCh38/ hg38	ENSG00000181291
TSPEAR	GRCh38/ hg38	ENSG00000175894
WBP2	GRCh38/ hg38	ENSG00000132471

Table S2. Research panel

Gene name	Ref. Genome	Ensemble no.
CDC42	GRCh37/ hg19	ENSG00000070831
ABCC1	GRCh37/ hg19	ENSG00000124574
CDC14A	GRCh37/ hg19	ENSG00000079335
CD164	GRCh37/ hg19	ENSG00000135535
ABHD12	GRCh37/ hg19	ENSG00000100997
CCDC50	GRCh37/ hg19	ENSG00000152492
ACTG1	GRCh37/ hg19	ENSG00000184009
CD151	GRCh37/ hg19	ENSG00000177697
COL9A3	GRCh37/ hg19	ENSG00000092758
ADCY1	GRCh37/ hg19	ENSG00000164742
ADGRV1	GRCh37/ hg19	ENSG00000164199
AIFM1	GRCh37/ hg19	ENSG00000156709
ALMS1	GRCh37/ hg19	ENSG00000116127
CRYM	GRCh37/ hg19	ENSG00000103316
AMMECR1	GRCh37/ hg19	ENSG00000101935
EDNRB	GRCh37/ hg19	ENSG00000136160
ELMOD3	GRCh37/ hg19	ENSG00000115459
EIF3F	GRCh37/ hg19	ENSG00000175390
FGF3	GRCh37/ hg19	ENSG00000186895
FKBP14	GRCh37/ hg19	ENSG00000106080
FGFR1	GRCh37/ hg19	ENSG00000077782
FGFR3	GRCh37/ hg19	ENSG00000068078
ANKH	GRCh37/ hg19	ENSG00000154122

GJA1	GRCh37/ hg19	ENSG00000152661
GLI3	GRCh37/ hg19	ENSG00000106571
GRAP	GRCh37/ hg19	ENSG00000154016
GPRASP2	GRCh37/ hg19	ENSG00000158301
GRXCR1	GRCh37/ hg19	ENSG00000215203
HMX2	GRCh37/ hg19	ENSG00000188816
HOMER2	GRCh37/ hg19	ENSG00000103942
HOXA1	GRCh37/ hg19	ENSG00000105991
KCNJ10	GRCh37/ hg19	ENSG00000177807
ARSG	GRCh37/ hg19	ENSG00000141337
KCNQ1	GRCh37/ hg19	ENSG00000053918
LARS2	GRCh37/ hg19	ENSG00000011376
Atoh1	GRCh37/ hg19	ENSG00000172238
MCM2	GRCh37/ hg19	ENSG00000073111
MPZ	GRCh37/ hg19	ENSG00000158887
ATP1A3	GRCh37/ hg19	ENSG00000105409
ATP2B2	GRCh37/ hg19	ENSG00000157087
ATP6N1B	GRCh37/ hg19	ENSG00000105929
NTN4	GRCh37/ hg19	ENSG00000074527
OPA1	GRCh37/ hg19	ENSG00000198836
OTOF	GRCh37/ hg19	ENSG00000115155
OTOA	GRCh37/ hg19	ENSG00000155719
OTOG	GRCh37/ hg19	ENSG00000188162
P2RX2	GRCh37/ hg19	ENSG00000187848
ATP6V1B1	GRCh37/ hg19	ENSG00000116039
PJVK	GRCh37/ hg19	ENSG00000204311
ATP6V1B2	GRCh37/ hg19	ENSG00000147416
BAIAP2L2	GRCh37/ hg19	ENSG00000128298
POLR1D	GRCh37/ hg19	ENSG00000186184
ROR1	GRCh37/ hg19	ENSG00000185483
BCS1L	GRCh37/ hg19	ENSG00000074582
BDP1	GRCh37/ hg19	ENSG00000145734
SERAC1	GRCh37/ hg19	ENSG00000122335
SIX1	GRCh37/ hg19	ENSG00000126778
SLC22A4	GRCh37/ hg19	ENSG00000197208
SOX9	GRCh37/ hg19	ENSG00000125398
SOBP	GRCh37/ hg19	ENSG00000112320
BSND	GRCh37/ hg19	ENSG00000162399
BTD	GRCh37/ hg19	ENSG00000169814
C10ORF2	GRCh37/ hg19	ENSG00000107815
CABP2	GRCh37/ hg19	ENSG00000167791
CACHD1	GRCh37/ hg19	ENSG00000158966
CACNA1D	GRCh37/ hg19	ENSG00000157388
USH1G	GRCh37/ hg19	ENSG00000182040
CATSPER2	GRCh37/ hg19	ENSG00000166762
CDH11	GRCh37/ hg19	ENSG00000140937
CDH23	GRCh37/ hg19	ENSG00000107736
CDK9	GRCh37/ hg19	ENSG00000136807
CDKN1C	GRCh37/ hg19	ENSG00000129757
CEACAM16	GRCh37/ hg19	ENSG00000213892
CEP250	GRCh37/ hg19	ENSG00000126001

CEP78	GRCh37/ hg19	ENSG00000148019
CHD7	GRCh37/ hg19	ENSG00000171316
CHSY1	GRCh37/ hg19	ENSG00000131873
CIB2	GRCh37/ hg19	ENSG00000136425
CISD2	GRCh37/ hg19	ENSG00000145354
CLDN14	GRCh37/ hg19	ENSG00000159261
CLDN9	GRCh37/ hg19	ENSG00000213937
CLIC5	GRCh37/ hg19	ENSG00000112782
CLPP	GRCh37/ hg19	ENSG00000125656
CLRN1	GRCh37/ hg19	ENSG00000163646
COCH	GRCh37/ hg19	ENSG00000100473
COL11A1	GRCh37/ hg19	ENSG00000060718
COL11A2	GRCh37/ hg19	ENSG00000204248
COL2A1	GRCh37/ hg19	ENSG00000139219
COL4A3	GRCh37/ hg19	ENSG00000169031
COL4A4	GRCh37/ hg19	ENSG00000081052
COL4A5	GRCh37/ hg19	ENSG00000188153
COL4A6	GRCh37/ hg19	ENSG00000197565
COL9A1	GRCh37/ hg19	ENSG00000112280
COL9A2	GRCh37/ hg19	ENSG00000049089
DCAF17	GRCh37/ hg19	ENSG00000115827
DCDC2	GRCh37/ hg19	ENSG00000146038
DFNA5	GRCh37/ hg19	ENSG00000105928
DFNB31	GRCh37/ hg19	ENSG00000095397
DFNB59	GRCh37/ hg19	ENSG00000204311
DFNB9	GRCh37/ hg19	ENSG00000115155
DIABLO	GRCh37/ hg19	ENSG00000184047
DIAPH1	GRCh37/ hg19	ENSG00000131504
DIAPH3	GRCh37/ hg19	ENSG00000139734
DLX5	GRCh37/ hg19	ENSG00000105880
DMXL2	GRCh37/ hg19	ENSG00000104093
DNAH5	GRCh37/ hg19	ENSG00000039139
DNAJC3	GRCh37/ hg19	ENSG00000102580
DNMT1	GRCh37/ hg19	ENSG00000130816
DPT	GRCh37/ hg19	ENSG00000143196
DSPP	GRCh37/ hg19	ENSG00000152591
DTNA	GRCh37/ hg19	ENSG00000134769
EDN3	GRCh37/ hg19	ENSG00000124205
EPS8	GRCh37/ hg19	ENSG00000151491
EPS8L2	GRCh37/ hg19	ENSG00000177106
ERAL1	GRCh37/ hg19	ENSG00000132591
ESPN	GRCh37/ hg19	ENSG00000187017
ESRP1	GRCh37/ hg19	ENSG00000104413
ESRRB	GRCh37/ hg19	ENSG00000119715
EXOSC2	GRCh37/ hg19	ENSG00000130713
EYA1	GRCh37/ hg19	ENSG00000104313
EYA4	GRCh37/ hg19	ENSG00000112319
FAM136A	GRCh37/ hg19	ENSG00000035141
FAM65B	GRCh37/ hg19	ENSG00000111913
FDXR	GRCh37/ hg19	ENSG00000161513
FGFR2	GRCh37/ hg19	ENSG00000066468

FITM2	GRCh37/ hg19	ENSG00000197296
FOXC1	GRCh37/ hg19	ENSG00000054598
FOXF2	GRCh37/ hg19	ENSG00000137273
FOXI1	GRCh37/ hg19	ENSG00000168269
GAB1	GRCh37/ hg19	ENSG00000109458
GATA3	GRCh37/ hg19	ENSG00000107485
GIPC3	GRCh37/ hg19	ENSG00000179855
GJB2	GRCh37/ hg19	ENSG00000165474
GJB3	GRCh37/ hg19	ENSG00000188910
GJB6	GRCh37/ hg19	ENSG00000121742
GPR98	GRCh37/ hg19	ENSG00000121742
GPSM2	GRCh37/ hg19	ENSG00000121957
GRHL2	GRCh37/ hg19	ENSG00000083307
GRXCR2	GRCh37/ hg19	ENSG00000204928
GSDME	GRCh37/ hg19	ENSG00000105928
HARS	GRCh37/ hg19	ENSG00000170445
HARS2	GRCh37/ hg19	ENSG00000112855
HGF	GRCh37/ hg19	ENSG00000019991
HOXA2	GRCh37/ hg19	ENSG00000105996
HOXB1	GRCh37/ hg19	ENSG00000120094
HSD17B4	GRCh37/ hg19	ENSG00000133835
IARS2	GRCh37/ hg19	ENSG00000067704
IFNLR1	GRCh37/ hg19	ENSG00000185436
IGF1	GRCh37/ hg19	ENSG00000017427
IKZF2	GRCh37/ hg19	ENSG00000030419
ILDR1	GRCh37/ hg19	ENSG00000145103
KARS	GRCh37/ hg19	ENSG00000065427
KARS1	GRCh37/ hg19	ENSG00000065427
KCNE1	GRCh37/ hg19	ENSG00000180509
KCNQ4	GRCh37/ hg19	ENSG00000117013
KIT	GRCh37/ hg19	ENSG00000157404
KITLG	GRCh37/ hg19	ENSG00000049130
LHFPL5	GRCh37/ hg19	ENSG00000197753
LMX1A	GRCh37/ hg19	ENSG00000162761
LOXHD1	GRCh37/ hg19	ENSG00000167210
LRP2	GRCh37/ hg19	ENSG00000081479
LRTOMT	GRCh37/ hg19	ENSG00000284922
MAN2B1	GRCh37/ hg19	ENSG00000104774
MANBA	GRCh37/ hg19	ENSG00000109323
MARVELD2	GRCh37/ hg19	ENSG00000152939
MET	GRCh37/ hg19	ENSG00000105976
MGP	GRCh37/ hg19	ENSG00000111341
MIA3	GRCh37/ hg19	ENSG00000154305
MIR96	GRCh37/ hg19	ENSG00000199158
MITF	GRCh37/ hg19	ENSG00000187098
MPZL2	GRCh37/ hg19	ENSG00000149573
MSRB3	GRCh37/ hg19	ENSG00000174099
MT-ATP6	GRCh37/ hg19	ENSG00000198899
MT-ATP8	GRCh37/ hg19	ENSG00000228253
MT-CO1	GRCh37/ hg19	ENSG00000198804
MT-CO2	GRCh37/ hg19	ENSG00000198712

MT-CO3	GRCh37/ hg19	ENSG00000198938
MT-CYB	GRCh37/ hg19	ENSG00000198727
MT-ND1	GRCh37/ hg19	ENSG00000198888
MT-ND2	GRCh37/ hg19	ENSG00000198763
MT-ND3	GRCh37/ hg19	ENSG00000198840
MT-ND4	GRCh37/ hg19	ENSG00000198886
MT-ND4L	GRCh37/ hg19	ENSG00000212907
MT-ND5	GRCh37/ hg19	ENSG00000198786
MT-ND6	GRCh37/ hg19	ENSG00000198695
MT-RNR1	GRCh37/ hg19	ENSG00000211459
MT-RNR2	GRCh37/ hg19	ENSG00000210082
MT-TA	GRCh37/ hg19	ENSG00000210127
MT-TC	GRCh37/ hg19	ENSG00000210140
MT-TD	GRCh37/ hg19	ENSG00000210154
MT-TE	GRCh37/ hg19	ENSG00000210194
MT-TF	GRCh37/ hg19	ENSG00000210049
MT-TG	GRCh37/ hg19	ENSG00000210164
MT-TH	GRCh37/ hg19	ENSG00000210176
MT-TI	GRCh37/ hg19	ENSG00000210100
MT-TK	GRCh37/ hg19	ENSG00000210156
MT-TL1	GRCh37/ hg19	ENSG00000209082
MT-TL2	GRCh37/ hg19	ENSG00000210191
MT-TM	GRCh37/ hg19	ENSG00000210112
MT-TN	GRCh37/ hg19	ENSG00000210135
MT-TP	GRCh37/ hg19	ENSG00000210196
MT-TQ	GRCh37/ hg19	ENSG00000210107
MT-TR	GRCh37/ hg19	ENSG00000210174
MT-TS1	GRCh37/ hg19	ENSG00000210151
MT-TS2	GRCh37/ hg19	ENSG00000210184
MT-TT	GRCh37/ hg19	ENSG00000210195
MT-TV	GRCh37/ hg19	ENSG00000210077
MT-TW	GRCh37/ hg19	ENSG00000210117
MT-TY	GRCh37/ hg19	ENSG00000210144
MYH14	GRCh37/ hg19	ENSG00000105357
MYH9	GRCh37/ hg19	ENSG00000100345
MYO15A	GRCh37/ hg19	ENSG00000091536
MYO1A	GRCh37/ hg19	ENSG00000166866
MYO3A	GRCh37/ hg19	ENSG00000095777
MYO6	GRCh37/ hg19	ENSG00000196586
MYO7A	GRCh37/ hg19	ENSG00000137474
NARS2	GRCh37/ hg19	ENSG00000137513
NCOA3	GRCh37/ hg19	ENSG00000124151
NDP	GRCh37/ hg19	ENSG00000124479
NLRP3	GRCh37/ hg19	ENSG00000162711
NMNAT1	GRCh37/ hg19	ENSG00000173614
OSBPL2	GRCh37/ hg19	ENSG00000130703
OTOGL	GRCh37/ hg19	ENSG00000165899
PAX2	GRCh37/ hg19	ENSG00000075891
PAX3	GRCh37/ hg19	ENSG00000135903
PBX1	GRCh37/ hg19	ENSG00000185630
PCDH15	GRCh37/ hg19	ENSG00000150275

PDE1C	GRCh37/ hg19	ENSG00000154678
PDSS1	GRCh37/ hg19	ENSG00000148459
PDZD7	GRCh37/ hg19	ENSG00000186862
PEX1	GRCh37/ hg19	ENSG00000127980
PEX26	GRCh37/ hg19	ENSG00000215193
PEX6	GRCh37/ hg19	ENSG00000124587
PHEX	GRCh37/ hg19	ENSG00000102174
PHYH	GRCh37/ hg19	ENSG00000107537
PIP4P1	GRCh37/ hg19	ENSG00000165782
PISD	GRCh37/ hg19	ENSG00000241878
PLS1	GRCh37/ hg19	ENSG00000120756
PNPT1	GRCh37/ hg19	ENSG00000138035
POLD1	GRCh37/ hg19	ENSG00000062822
POLR1C	GRCh37/ hg19	ENSG00000171453
POU3F4	GRCh37/ hg19	ENSG00000196767
POU4F3	GRCh37/ hg19	ENSG00000091010
PPIP5K2	GRCh37/ hg19	ENSG00000145725
PRKCB	GRCh37/ hg19	ENSG00000166501
PRPS1	GRCh37/ hg19	ENSG00000147224
PRRX1	GRCh37/ hg19	ENSG00000116132
PSIP1	GRCh37/ hg19	ENSG00000164985
PTPRQ	GRCh37/ hg19	ENSG00000139304
PTPRS	GRCh37/ hg19	ENSG00000105426
RDX	GRCh37/ hg19	ENSG00000137710
REST	GRCh37/ hg19	ENSG00000084093
RIPOR2	GRCh37/ hg19	ENSG00000111913
RMND1	GRCh37/ hg19	ENSG00000155906
RPS6KA3	GRCh37/ hg19	ENSG00000177189
S1PR2	GRCh37/ hg19	ENSG00000267534
SALL1	GRCh37/ hg19	ENSG00000103449
SALL4	GRCh37/ hg19	ENSG00000101115
SCD5	GRCh37/ hg19	ENSG00000145284
SDHD	GRCh37/ hg19	ENSG00000204370
SEMA3D	GRCh37/ hg19	ENSG00000153993
SEMA3E	GRCh37/ hg19	ENSG00000170381
SERPINB6	GRCh37/ hg19	ENSG00000124570
SGO2	GRCh37/ hg19	ENSG00000163535
SIX5	GRCh37/ hg19	ENSG00000177045
SLC12A2	GRCh37/ hg19	ENSG00000064651
SLC17A8	GRCh37/ hg19	ENSG00000179520
SLC19A2	GRCh37/ hg19	ENSG00000117479
SLC26A4	GRCh37/ hg19	ENSG00000091137
SLC26A5	GRCh37/ hg19	ENSG00000170615
SLC29A3	GRCh37/ hg19	ENSG00000198246
SLC33A1	GRCh37/ hg19	ENSG00000169359
SLC44A4	GRCh37/ hg19	ENSG00000204385
SLC4A11	GRCh37/ hg19	ENSG00000088836
SLC52A2	GRCh37/ hg19	ENSG00000185803
SLC52A3	GRCh37/ hg19	ENSG00000101276
SLITRK6	GRCh37/ hg19	ENSG00000184564
SMAD4	GRCh37/ hg19	ENSG00000141646

SMPX	GRCh37/ hg19	ENSG00000091482
SNAI2	GRCh37/ hg19	ENSG00000019549
SOX10	GRCh37/ hg19	ENSG00000100146
SPATA5	GRCh37/ hg19	ENSG00000145375
SPNS2	GRCh37/ hg19	ENSG00000183018
STAG2	GRCh37/ hg19	ENSG00000101972
STIM1	GRCh37/ hg19	ENSG00000167323
STRC	GRCh37/ hg19	ENSG00000242866
SUCLA2	GRCh37/ hg19	ENSG00000136143
SUCLG1	GRCh37/ hg19	ENSG00000163541
SYNE4	GRCh37/ hg19	ENSG00000181392
SYT2	GRCh37/ hg19	ENSG00000143858
TARS2	GRCh37/ hg19	ENSG00000143374
TBC1D24	GRCh37/ hg19	ENSG00000162065
TBL1X	GRCh37/ hg19	ENSG00000101849
TCOF1	GRCh37/ hg19	ENSG00000070814
TECTA	GRCh37/ hg19	ENSG00000109927
TFAP2A	GRCh37/ hg19	ENSG00000137203
TIMM8A	GRCh37/ hg19	ENSG00000126953
TJP2	GRCh37/ hg19	ENSG00000119139
TMC1	GRCh37/ hg19	ENSG00000165091
TMEM132E	GRCh37/ hg19	ENSG00000181291
TMEM59	GRCh37/ hg19	ENSG00000116209
TMIE	GRCh37/ hg19	ENSG00000181585
TMPRSS3	GRCh37/ hg19	ENSG00000160183
TMTC2	GRCh37/ hg19	ENSG00000179104
TNC	GRCh37/ hg19	ENSG00000041982
TNFRSF11B	GRCh37/ hg19	ENSG00000164761
TOP2B	GRCh37/ hg19	ENSG00000077097
TPRN	GRCh37/ hg19	ENSG00000176058
TRIOBP	GRCh37/ hg19	ENSG00000100106
TRMU	GRCh37/ hg19	ENSG00000100416
TRNT1	GRCh37/ hg19	ENSG00000072756
TRRAP	GRCh37/ hg19	ENSG00000196367
TSHZ1	GRCh37/ hg19	ENSG00000179981
TSPEAR	GRCh37/ hg19	ENSG00000175894
TUBB4B	GRCh37/ hg19	ENSG00000188229
TWNK	GRCh37/ hg19	ENSG00000107815
TYR	GRCh37/ hg19	ENSG00000077498
USH1C	GRCh37/ hg19	ENSG00000006611
USH2A	GRCh37/ hg19	ENSG00000042781
VCAN	GRCh37/ hg19	ENSG00000038427
WBP2	GRCh37/ hg19	ENSG00000132471
WFS1	GRCh37/ hg19	ENSG00000109501
WHRN	GRCh37/ hg19	ENSG00000095397
XYLT2	GRCh37/ hg19	ENSG00000015532
YAP1	GRCh37/ hg19	ENSG00000137693
ABHD12	GRCh37/ hg19	ENSG00000100997
ACOX1	GRCh37/ hg19	ENSG00000161533
ALMS1	GRCh37/ hg19	ENSG00000116127
AP1S1	GRCh37/ hg19	ENSG00000106367

ATP11A	GRCh37/ hg19	ENSG00000068650
BMP4	GRCh37/ hg19	ENSG00000125378
CAT	GRCh37/ hg19	ENSG00000121691
CDC6	GRCh37/ hg19	ENSG00000094804
CDT1	GRCh37/ hg19	ENSG00000167513
CEMIP	GRCh37/ hg19	ENSG00000103888
CLRN2	GRCh37/ hg19	ENSG00000249581
COG4	GRCh37/ hg19	ENSG00000103051
CRLS1	GRCh37/ hg19	ENSG00000088766
DHODH	GRCh37/ hg19	ENSG00000102967
DLL1	GRCh37/ hg19	ENSG00000198719
EDNRA	GRCh37/ hg19	ENSG00000151617
EFTUD2	GRCh37/ hg19	ENSG00000108883
EIF4A3	GRCh37/ hg19	ENSG00000141543
FGF10	GRCh37/ hg19	ENSG00000070193
FOXI3	GRCh37/ hg19	ENSG00000214336
FRAS1	GRCh37/ hg19	ENSG00000138759
FREM2	GRCh37/ hg19	ENSG00000150893
GDF6	GRCh37/ hg19	ENSG00000156466
GGPS1	GRCh37/ hg19	ENSG00000152904
GNAI3	GRCh37/ hg19	ENSG00000065135
GREB1L	GRCh37/ hg19	ENSG00000141449
GRIP1	GRCh37/ hg19	ENSG00000155974
GSC	GRCh37/ hg19	ENSG00000133937
HAAO	GRCh37/ hg19	ENSG00000162882
HMX1	GRCh37/ hg19	ENSG00000215612
HSPA9	GRCh37/ hg19	ENSG00000113013
KCNJ16	GRCh37/ hg19	ENSG00000153822
KDM3B	GRCh37/ hg19	ENSG00000120733
KDM6A	GRCh37/ hg19	ENSG00000147050
KMT2D	GRCh37/ hg19	ENSG00000167548
LETM1	GRCh37/ hg19	ENSG00000168924
MASP1	GRCh37/ hg19	ENSG00000127241
MN1	GRCh37/ hg19	ENSG00000169184
MORC2	GRCh37/ hg19	ENSG00000133422
NRN1	GRCh37/ hg19	ENSG00000124785
OFD1	GRCh37/ hg19	ENSG00000046651
OGDHL	GRCh37/ hg19	ENSG00000197444
ORC1	GRCh37/ hg19	ENSG00000085840
ORC4	GRCh37/ hg19	ENSG00000115947
ORC6	GRCh37/ hg19	ENSG00000091651
OTX2	GRCh37/ hg19	ENSG00000165588
OXR1	GRCh37/ hg19	ENSG00000164830
PLCB4	GRCh37/ hg19	ENSG00000101333
PMP22	GRCh37/ hg19	ENSG00000109099
POLR1A	GRCh37/ hg19	ENSG00000068654
RNF220	GRCh37/ hg19	ENSG00000187147
RPS28	GRCh37/ hg19	ENSG00000233927
SF3B4	GRCh37/ hg19	ENSG00000143368
SGPL1	GRCh37/ hg19	ENSG00000166224
SLC17A1	GRCh37/ hg19	ENSG00000124568

SOX2	GRCh37/ hg19	ENSG00000181449
SPATA5L1	GRCh37/ hg19	ENSG00000171763
SPATC1L	GRCh37/ hg19	ENSG00000160284
SPTBN4	GRCh37/ hg19	ENSG00000160460
STX4	GRCh37/ hg19	ENSG00000103496
STXBP3	GRCh37/ hg19	ENSG00000116266
THOC1	GRCh37/ hg19	ENSG00000079134
USP48	GRCh37/ hg19	ENSG00000090686
YARS1	GRCh37/ hg19	ENSG00000134684
